# The quality of health services provided to remote dwelling aboriginal infants in the top end of northern Australia following health system changes: a qualitative analysis

**DOI:** 10.1186/s12887-017-0849-1

**Published:** 2017-03-31

**Authors:** Cathryn M. Josif, Sue Kruske, Sue V. Kildea, Lesley M. Barclay

**Affiliations:** 1 University Centre for Rural Health Sydney School of Public Health, University of Sydney, Sydney, NSW 2480 Australia; 2 School of Nursing Midwifery and Social Work, The University of Queensland (UQ), Brisbane, 4010 Australia

**Keywords:** Aboriginal, Anaemia, Australia, Child Health, Growth faltering, Indigenous, Infant, Remote

## Abstract

**Background:**

In Australia the health outcomes of remote dwelling Aboriginal infants are comparable to infants in developing countries. This research investigates service quality, from the clinicians’ perspective and as observed and recorded by the researcher, in two large Aboriginal communities in the Top End of northern Australia following health system changes.

**Methods:**

Data were collected from semi-structured interviews with 25 clinicians providing or managing child health services in the two study sites. Thirty hours of participant observation was undertaken in the ‘baby-rooms’ at the two remote health centres between June and December 2012. The interview and observational data, as well as field notes were integrated and analysed thematically to explore clinicians’ perspectives of service delivery to infants in the remote health centres.

**Results:**

A range of factors affecting the quality of care, mostly identified before health system changes were instigated, persisted. These factors included ineffective service delivery, inadequate staffing and culturally unsafe practices. The six themes identified in the data: *‘very adhoc’*, *‘swallowed by acute’*, *‘going under’*, *‘a flux’*, *‘a huge barrier’* and *‘them and us’* illustrate how these factors continue, and when combined portray a *‘very chaotic system’*.

**Conclusion:**

Service providers perceived service provision and quality to be inadequate, despite health system changes. Further work is urgently needed to improve the quality, cultural responsiveness and effectiveness of services to this population.

**Electronic supplementary material:**

The online version of this article (doi:10.1186/s12887-017-0849-1) contains supplementary material, which is available to authorized users.

## Background

Health disparities between Aboriginal and non-Aboriginal women and infants are well established [1] and exacerbated by remoteness [2]. Despite a decline in Australian Aboriginal infant mortality, the rate is still twice that of non-Aboriginal infants [1]. Twice as many Aboriginal infants are low birth weight (LBW) (≤ 2500 g) and a greater proportion are born preterm when compared with non-Aboriginal infants [1].

Remote Health Centre (RHC) attendance in the Northern Territory (NT) of Australia by remote-dwelling Aboriginal infants in the first year of life is high, with presentations predominantly for respiratory, skin and gastrointestinal symptoms [3]. These infants also have a high hospitalisation rate in their first year of life [3]. Malnutrition, anaemia and acute illness are widespread in this population, starting early, and persisting throughout the first year of life [4].

Strong evidence links events in a child’s early years with adult health and wellbeing [5, 6]. Targeted interventions and culturally appropriate high-quality health care in the early years can reduce rates of complex chronic disease from infancy through to adulthood [7, 8], particularly in disadvantaged populations [9].

Most Australians can access well-established ‘well-child’ health programs, with child health and developmental checks provided by qualified child and family health nurses (CFHNs)^1^ These are registered nurses and/or midwives with additional postgraduate qualifications and experience in child and family health nursing who ‘promote child development, maternal and family capacity, and provide services for both primary and secondary prevention of physical and psychosocial issues’ [10] [p200].

‘Well-child’ and family checks are provided via a schedule of regular contacts with children between the ages of 0–5 years that correspond with critical developmental stages [11]. Targeted interventions are offered to children and families with identified needs to actively manage concerns promptly and circumvent the need for further referral [11]. Although a ‘well-child’ health service has been available in Australia for over 100 years [10], this service, promoted and provided by qualified staff, is not routinely available to remote dwelling infants.

The 1 + 1 = A Healthy Start to Life Study was a five year (2007–2012) mixed-methods study using participatory action research (PAR) approaches and funded by the Australian National Health and Medical Research Council and Australian Research Council. The study was developed in response to concerns expressed by Aboriginal women, policy makers and clinicians about the quality of maternal and infant health services in the Top End (TE) of the NT [12] and previously documented in NT publications and reports [13–15]. Each sub-study within the Healthy Start to Life study was conceived, planned, conducted and reported with the primary aim of service improvement [16] and several focused on infants [3, 4, 17]. The infant sub-studies provided a snapshot of the overall quality of health service delivery prior to infant service reforms [16].

### The Northern Territory infant health service reforms

Data from the Healthy Start to Life investigations into maternal and infant health (MIH) care were presented to Department of Health (DoH) leaders, policy makers and clinicians in 2008/2009. A number of reforms to the remote-dwelling infant health service were subsequently undertaken. Some of these reforms occurred following the data feedback, and others were independent of the research. These reforms included; implementation of the Healthy Under 5 Kids (HU5K) program and an education package to support staff to deliver this program. Designated CFHN and Aboriginal Community Worker (ACW) positions were also established in the two Healthy Start to life study sites.

#### Healthy under 5 kids program

The new HU5K Program, introduced in 2009, aimed to shift the focus of primary health care delivery to the 0-5 year old population from what had been selective primary health care to a comprehensive primary health care model [18].This program extended the DoH Growth Assessment and Action Program, the previous mechanism for detecting anaemia and child growth and interventions for growth faltering [18]. The HU5K program encompassed age-specific child health surveillance, including growth assessment and childhood vaccinations [19]. The six visits scheduled between birth and twelve months of age were designed to be opportunities for health promotion and education. The assessments at different growth stages focused on; bonding and attachment, response to caregiver concerns, developmental milestones, living conditions, nutrition and the introduction of solids [19].

#### The Healthy under 5 kids education package

In 2009 an education package was jointly developed by the DoH and the regional university to support delivery of the HU5K program. Targeting RHC staff without formal CFHN qualifications, the package comprised eight modules incorporating: primary health care, child development and assessment tools, physical and cognitive aspects of growth and development, social and emotional development, psychosocial aspects of parenting, working in partnership with parents, brief intervention and the HU5K schedule [20]. This stand-alone, self-directed education package took approximately 20 h to complete and upon completion, doctors and nurses who completed the package were eligible for credit towards tertiary education programs. Completion of the package was not a pre-requisite for staff working with infants, although these staff were strongly encouraged and supported to do it.

#### Child and family health nurse and Aboriginal community worker positions

In 2010 four designated fulltime equivalent (FTE) CFHN positions were established in the two study sites (two FTE in each community) and two part-time ACW positions (one position in each community). The ACW position is a non-clinical role, undertaking health promotion, cultural brokerage and community development [21] to complement the role of clinicians in RHCs. The ACW (child health) position was established to carry out the above functions, and work in partnership with the CFHNs, to increase awareness of the importance of the early years and promote the HU5K program.

The aim of this paper is to report the qualitative findings of a sub-study exploring the quality of service provision to remote-dwelling Aboriginal infants, from a clinician’s perspective, after the above reforms were instigated. Quantitative findings will be reported elsewhere.

### Setting

Of the NT’s population of 244,000 people [22], 30% are estimated to be Aboriginal and Torres Strait Islander [23] with one quarter (27%) of the Aboriginal population living in remote or very remote locations [24]. Children under 15 years of age comprise one-fifth of Australia’s total population and the NT has the highest proportion of children (23%). One quarter (26%) of these dwell in ‘outback areas’ [25].

The study sites were the RHCs in two remotely located Aboriginal communities, A and B (populations between 2000 and 2500). The communities are situated 500 km in opposite directions from the regional centre; the location of the major referral hospital.

At the time of the study the RHCs were open during business hours with staff rostered ‘on call’ for after-hours presentations. Care was provided by doctors, remote area nurses (RANs) who may also be registered midwives, remote area midwives, Aboriginal Health Workers (AHWs) and ACWs. Unlike ACWs who assume a non-clinical role, AHWs primarily assume a clinical role and other responsibilities as required, including administration, clinical, health promotion and education. Like ACWs, they are also cultural brokers providing a link between traditional and Western beliefs [26].

Despite available funding for two CFHNs in each community, and attempts to employ qualified CFHNs, recruitment and retention to these positions proved difficult. No dedicated CFHNs had been employed. Community A had employed a registered nurse/midwife, who was undertaking her post-graduate CFHN qualifications, on a fly in fly out basis, three days per week. She left after seven months for personal reasons and was not replaced during the data collection period. Community B recruited a qualified CFHN who resigned after six weeks. The reasons for her departure were unclear and she was unavailable for interview. This position had not been recruited to during the data collection period, although a RAN with CFHN qualifications worked a month on month off roster. A CFHN, based in the regional centre, visited the community for 2–3 days each fortnight to provide a well-child health program outside of the RHC. No ACWs were employed to work in partnership with the CFHNs in either community during the data collection period.

Recruitment and retention issues pertained to; a lack of community staff housing, absence of clinical governance to support the CFHN roles, poor management and collegial support and discord regarding expectations of the CFHN (well-child) role compared with a ‘paediatric’ nursing (ill child) role.

Situated within the RHC and staffed by one or two RANs were rooms assigned to the 0–5 years population commonly called ‘baby-rooms’. These rooms contained all the equipment required for working with infants, for example measuring mats, infant scales, immunisation fridge etc. Health promotion posters aimed at Aboriginal parents and infants targeting eye health, nutrition and hygiene, for example, were displayed. There were also child-friendly murals, mobiles and pictures using both western and Aboriginal imagery. While the baby-rooms were similar in both RHCs, the organisation and delivery of infant health services differed slightly, as described below.

In RHC A, one RAN worked in the baby-room solely to provide follow-up for infants with growth faltering and anaemia. As well as being on-call for general (not just paediatric) after-hours presentations, this RAN held other considerable responsibilities in the RHC, for example ordering pharmacy, palliative care or mental health and consequently was often unavailable to work in the ‘baby-room’. The ‘well-child’ program and immunisations were completed one day per week by a GP paediatrician and another RAN. Acute infant care was provided by other RHC clinicians as required.

In RHC B two RANs worked in the baby-room and every child who presented at this RHC was directed to the baby-room. As such, these RANs were expected to complete the ‘well-child’ program, immunisations, and provide acute care. These two RANs also worked on call for general after-hours presentations and were allocated other RHC portfolios. Like their counterpart in RHC A they were subsequently not always available to work in the baby-room.

Both study sites used an electronic health record system (EHR). While this EHR interfaced with other electronic databases, for example the NT Childhood Immunisation Register and the NT Rheumatic Heart Disease Register, it did not always interface with EHR systems used in non-DoH communities [27]. All RHC staff were expected to follow treatment guidelines developed and endorsed by the NT DoH^2^ [28]. For example, prompts were generated for the well-child program within the EHR. Infants with abnormal findings e.g. anaemia and growth faltering requiring specific treatment and follow-up were meant to be commenced on ‘action plans’ generated by clinicians as needed.

Paediatricians visited each community monthly. Qualified CFHNs from the regional centre provided an outreach service and visited every four to six weeks to provide support and guidance to the RANs who were commonly without child health qualifications [29]. These CFHNs advised on growth and development, immunisations and health promoting activities for infants and their families. They did not directly deliver services.

## Methods

### Design

The overarching Healthy Start to Life study used mixed-methods sub-studies and a participatory approach to explore aspects of MIH care for remote dwelling women and infants. [16]. The aim of this sub-study is to investigate service quality provided to infants in RHCs, from the clinicians’ perspective and as observed by the researcher, following health system changes.

The study reported here was added to the broader study, to help evaluate problems identified in the earlier research concerning quality of care provision and the impact of attempted improvements. The overarching study was designed using a pragmatic approach considered ‘a practical and outcome oriented method of inquiry’ [30] [p 17]. This study combined a retrospective cohort study of the records of Aboriginal infants born September 2009 – June 2011 (*n* = 232) and 25 clinician interviews, plus 30 h of observational data undertaken in the two study sites between June and December 2012. This paper reports the interview and observational findings only.

### Participants

Participants *(n = 25)* who provided care for infants and their families in the two study sites (or visited in an outreach capacity) were purposefully recruited by the first author who made initial contact by telephone and email One person did not respond to the invitation for interview. All were female and DoH employees. Only one employee was Aboriginal, a reflection of DoH employment figures where Aboriginal people account for only 1% of the total workforce [31]. The participants, as presented in Table 1, had worked in their current roles between two months and 12 years.

**Table 1:** Participant characteristics

Location and Occupation	Total (*n* = 25)
*Health Centre A & B (n = 15)*	1
Midwife	4
District medical officer	2
RAN	4
CMHN	1
GP Paediatrician	1
Health Centre Manager	3
*Regional hospital (n = 3)*	
Midwife	1
GP Paediatrician	2
*Outreach services (n = 7)*	
Midwife	2
CMHN	3
Diabetic educator	1
Strong Woman Coordinator	1

*RAN = Remote Area Nurse, CMHN = Child*, *And Maternal Health Nurse, GP = General*, *Practitioner*

#### Interviews

Interview prompts explored clinicians’ perspectives of service delivery. Questions related to the strengths of the infant health service, the quality of service delivery to infants and the challenges of providing health care in the study sites. A semi-structured in-depth approach was used allowing flexibility and exploration in more detail of the views and experiences of participants. [32] Most interviews were conducted face-to-face by the first author, in locations chosen by the participants, for example their individual offices (*n* = 23) and two by telephone.

All participants consented to the audio recording of their interviews, which lasted 30–90 min. All interviews were transcribed verbatim, 10 by the first author and 15 by a professional transcriber (with content verified by the first author). While there is considerable debate regarding the advantages or otherwise of sharing interview transcripts with participants [33, 34] it recognises participant rights and strengthens the relationship between researcher and participant [33]. All the participants in this sub-study were emailed a copy of their interview transcripts and given two weeks to comment or make amendments. No changes were suggested by participants or made.

Given the pragmatic design of the study a practical, yet innovative approach to determine sample size was used. Information Power suggests ‘that the more information the sample holds, relevant for the actual study, the lower amount of participants is needed’ [35]. Using this approach the sample size required to achieve Information Power in this study was not theory driven. But a combination of the study aim, sample specificity, use of established theory, dialogue quality and analysis strategy [35].

#### Observational data

While the semi-structured interviews explored clinicians’ perspectives, participant observation (30 h) undertaken by the first author allowed for direct observation of service provision by clinicians in the baby-rooms of the two RHCs (fifteen hours in each). A structured observation checklist of service provision used in the baseline study [36] was used in this sub-study, for consistency and to minimise bias, ensuring that only directly observable events, and not interpretations were recorded. Participant observation allowed the researcher to better understand other qualitative data and reinforce the trustworthiness of the findings. Field notes were recorded, during and following observations to aid in describing the environment, behaviours and verbal and non-verbal communication between clinicians and clients. Observational data and field notes were transcribed verbatim.

#### Reflexivity and researcher identity

The first author has previously explored reflexivity and her researcher identity as part of the 1 + 1 Study observing that as a researcher she is inextricably linked to her previous and life experiences and that these in turn influence how she interacts with all aspects of the research process [37]. She is a white, middle class woman who is a midwife and child health nurse who has worked as a RAN in Aboriginal communities. The first author was aware of the ethical implications of working as a participant observer and recorded this in reflections on her fieldwork.

*I tell people I am a CHN, midwife and that I have worked as a RAN. I believe this gives me something in common with the participants. It puts me on the same page and I am not perceived as an expert with no experience of living and working in remote communities who is coming in only to criticise and find fault (Field notes).*

### Data analysis

The transcribed data (interviews, observational data and field notes) were combined and analysed thematically to substantiate and strengthen the analysis [38]. The first author completed a preliminary thematic analysis ascribing meaning to the data and generating categories and subcategories, for the most part using participants’ own language. The categories most often mentioned were highlighted and similarities and dissimilarities recorded. The categories were then grouped together and themes and subthemes emerged. These were shared with the second and fifth authors, who were both familiar with the setting; for review and modification as needed. Total consensus resulted when no data changes or refinements were required. Data were stored and organised for analysis using NVIVO 9 (™ QSR International).

## Results

**Fig. 1:**
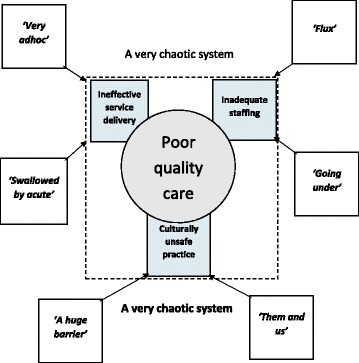
A very chaotic system. Themes and subthemes

Six subthemes were identified in the data using participant language. Considered together these subthemes contributed to the overarching perception of infant care occurring within what one participant described as ‘a *very chaotic system’*. The subthemes were further grouped into three broad themes to show how each contributed to ineffective service delivery, inadequate staffing and culturally unsafe practice and consequently poor quality care. See Fig. 1. These themes will now be discussed in full.

### Ineffective service delivery

Ineffective service delivery is exemplified by two themes (*‘very adhoc’* and *‘swallowed by acute’)*. These themes illustrate inadequate practices related to hospital discharge processes, non-adherence to treatment guidelines and an emphasis on acute care, to the detriment of the well-child program.

#### ‘Very adhoc’

*‘Very adhoc’* (P 4) relates to the poor quality and often inaccurate or absent discharge information regarding hospitalised infants returning to their community, from the regional hospital to the RHC and the adhoc adherence to treatment guidelines by some RHC staff. The transfer of electronic discharge information from hospital to RHC was described as *‘abysmal’* (P22) *‘suboptimal’* (P23) and ‘*in the too hard basket’* (P6). The regional hospital policy was for discharge summaries to be completed and forwarded directly to the infant’s EHR within 48 h of discharge. However, all participants described how discharge summaries frequently did not arrive, with one participant commenting *‘a discharge summary might turn up six months later and that’s hopeless’* (P6). When summaries did arrive they commonly contained inaccurate information or information was missing altogether. The poor transfer of information is typified in this abstract from research field notes.

*Nurse x and I were in the baby-room and she showed me the notes of an infant who had been evacuated to the regional hospital. The next entry in the infant’s record post evacuation was five weeks later. The infant had been discharged from hospital after nine days and had been back in the community for one month, but not been seen in the RHC. Medications prescribed on the discharge summary had not been administered. No admission or discharge weights were recorded in the summary (August 2012).*

Poor information transfer resulted in infants who were supposed to receive rigorous follow-up missing prescribed treatments. This system failure was described by a number of participants and best illustrated in the following narrative:

*Two [preterm] children were checked at six months … never [given pentavite or iron]. [These medications] didn’t appear in the discharge summary. Mum wasn’t given any [medication] to take home. Nurses [in the RHC] were unaware [of guidelines that preterm children required pentavite and iron]. So [these children] were profoundly anaemic … haemoglobins of 70 which is [is likely to affect] the child’s development and completely avoidable* (P22).

This incident also provides an example of how clinical guidelines were often not adhered to. In this instance, the staff were unaware of the clinical guidelines a result of poor preparation and training for the child health role. Ignorance of the guidelines was not always the cause of poor quality care. Participants referred to the adhoc way guidelines were adhered to and sometimes completely disregarded observing *‘there’s something about treatment guidelines that people … want to do their own thing, or they think they know better’* (P17) and clinicians *‘get their own agendas and they don’t really follow the Under Fives program like they should’* (P6).

Another participant noted that infants *‘get their haemoglobin checked and [are] identified [as] anaemic. But staff aren’t then treating [as per guidelines]. Clinicians [say] ‘oh well we’ll recheck it in a month’* (P10). This participant commented that *‘the quality of care is going to be related to staff going by the guidelines’* (P10) and believed that a potential explanation for why guidelines weren’t followed was because *‘people are used to seeing anaemia, it’s normalised … like we’re used to [LBW] infants. We still have to keep in mind that it’s not normal even if it is common* (P10).

#### ‘Swallowed by acute’

This theme describes how *‘acute [care] just swallows everything else’* (P4) and overwhelmed the well-child program which was subsequently *‘ignored … It’s not acute and it’s not seen as important so [it] doesn’t get done’* (P6).

The well-child health program appeared to be so devalued by some clinicians, that it was not seen as legitimate ‘work’. As one clinician working in the baby-room remarked *‘let me know if kids come in I am just going to pharmacy to do my real job’* (Field notes). One participant described how primary health care programs, including the well-child program, could be cancelled due to staff shortages. This participant remarked how *‘we were really short of staff [and] we were told there was no program work to be done today’* (P5). Participants also reported how previously a baby room had been closed by a RHC manager because *‘staff did not want to work [there]’* (P22) as it was *‘too noisy … too busy … pretty chaotic’* (P23). This action was perceived by one participant as an apparent attempt by the RHC manager to *‘eliminate the well-child program’* (P22). These examples, by participants also demonstrate the lack of recognition by RHC management of the well-child program as important work, despite it being DoH core business.

### Inadequate staffing

Inadequate staffing is demonstrated in the following two themes that illustrate the impact of staff ill prepared to work in the well-child program (*‘going under’*) and high staff turnover on the quality of care (*‘a flux’*).

#### 'Going Under'

Many of the staff who were working with infants in the RHC, while highly trained in acute care, lacked the requisite skills to deliver the well-child program and none had completed the HU5K educational package. Between 2009, when the package was introduced, and July 2011, there had been 192 enrolments by staff across the NT: 36 staff had completed the package, 27 were in progress and 130 had enrolled but not completed the package in the allocated timeframe (personal communication). As one participant with no child health, paediatric or midwifery experience explained *… [I had] done a [vaccination] course [so] the manager flung me in the baby room and said ‘there you go you can do the vaccinations and you can be the ‘baby-nurse’* (P19).

The quality of the care provided to infants was viewed as *‘dependent on the staff member … and their experience’* (P1). A participant reflected how *‘we’re all trained in accident and emergency or as coronary care or intensive care but its primary health care here so we really do need more training’* (P19). The effect of this on the quality of care was interpreted by one participant as *‘the reality is probably that it’s not a great quality at the moment because the positions aren’t filled with people who are qualified’* (P20).

Thus, well-child health assessments were infrequently or inappropriately completed because clinicians lacked appropriate skills and training to do them. This is supported by the following observation recorded in field notes ‘development checks that targeted two and four month old infants were completed on nine and 12 month old infants’. The lack of training and support also had a considerable detrimental impact upon staff morale. Many participants who had worked in the baby-rooms became emotional when relating their experiences to the researcher, with some becoming tearful. Despite support from the regionally located CFHNs they described how *‘you’re expected to do the job without the background, without the knowledge and that … is a massive stress’ (P20).* Many of them recounted their experiences using terms such as *‘drowning … going under’* (P20) and *‘sink or swim’* (P19).

#### ‘A flux’

The theme, *‘a flux’* (P17) pertains to high staff rotation through the baby-rooms. During the data collection period, a mix of permanent and locum staff rotated through the baby-rooms in both communities; for periods ranging from one week to one month. Staff were often reluctant to work in this room because it was perceived as a *‘hectic and demanding environment’* (Field notes) and staff rotation was identified as a strategy *‘to prevent stress and burnout’* (Field notes) by having staff work in the area for short lengths of time. While adopted as a strategy to prevent burnout it was considered by some participants that exposing *‘inexperienced and untrained staff to the pressure and stress of working in a specialised area wouldn’t happen elsewhere’* (Field notes).

The implications of this high staff rotation on quality of care were identified by participants as twofold. Firstly, it impacted on community members’ ability to establish rapport and relationships with the baby-room staff. Participants observed how it *‘makes it really difficult for the community because it takes them a long time to get to know you’* (P14) and *‘if [carers] don't know the person who's working in the baby-room they won't come* (P23). Secondly, the volume of staff moving through the baby-room resulted in inconsistent care. This was exemplified by a participant who observed that *‘so many people [move] through the baby room and they all set up different systems and different ways of working’* (Field notes).

### Culturally unsafe practice

Culturally unsafe practice is revealed in two themes (‘a huge barrier’ and ‘them and us’). These themes show how the negligible numbers of Aboriginal staff working in the RHCs and perceived racist behaviours by some staff are huge barriers to community engagement.

#### ‘A huge barrier’

This theme describes how the minimal numbers of Aboriginal staff working in the two RHCs was perceived by the participants as *‘a huge barrier to community members engaging with the RHC’* (P14). The crucial role of Aboriginal staff in providing a link with the community was acknowledged by most participants and best exemplified by the participant who observed.

*‘We are Balanda* 3 *people. We think in a different way and if people can talk to their own people that works much better. ... You can see people relax straight away … because there is an Aboriginal person there … people talk much more when they talk to Aboriginal people’* (P9).

While participants acknowledged the important role of Aboriginal staff, with one observing *‘a [RHC] is only as good as the [Aboriginal staff] that you’ve got’* (P5) only five Aboriginal staff were employed at the time of data collection, all in one community and none in child health roles. The researcher noted that *‘one AHW worked in chronic disease and two others worked in men’s health’* (Field notes). Two ACWs did ‘*a little bit of admin, a little bit of filing, a bit of driving, a bit of whatever’* (P1). Despite the availability of funding and being considered *‘imperative to the success of the [child health] program’* (P1) over the course of data collection no ACWs were recruited to the newly established positions to work in partnership with the CFHNs.

Participants reflected on a number of reasons why there were now so few Aboriginal staff working in the RHCs. They observed that Aboriginal staff ‘*haven’t really been valued in the past’* (P6) and postulated that high staff turnover and the increased use of agency staff, unused to working with Aboriginal staff, had resulted in Aboriginal workers being *‘pushed to one side*’ (P3).

#### ‘Them and us’

This theme reflects the perceptions of racist behaviours of some clinicians, who, as articulated by one participant *‘can make it like them and us’* (P12). Some participants believed racist attitudes were apparent in how Aboriginal caregivers were treated by some clinicians, with one participant expressing concern at how caregivers were spoken to. She commented ‘*I have [said to staff] you don’t speak to people like that … but they don’t want to know … it’s this attitude of I’m the boss …’* (P5). Another participant observed the consequence of this as people avoiding the RHC, remarking *‘you know if [mothers] are getting a negative kind of attitude and approach … it is really hard to get mothers to come’* (P1). An example of the negative attitude and approach experienced by some caregivers is shown in the Additional file 1 which describes an encounter between a RAN and a mother in a baby-room.

## Discussion

This study investigated the quality of services provided to remote-dwelling Aboriginal infants, from the clinicians’ perspective, and as observed by the researcher, following the launch of three initiatives designed to reform health services for remote dwelling infants. These were: 1) the redeveloped well-child health program, 2) an education package supporting the program and 3) establishment of dedicated CFHN and ACW positions in the two study sites.

A range of factors affecting the quality of care, identified before these initiatives were instigated remained problematic. These factors included ineffective service delivery, inadequate staffing and culturally unsafe practices [3, 4, 17, 39, 40]. The six themes identified and described in this paper illustrate how these factors persist and when combined portray a *‘very chaotic system’.*

Poor information transfer between the regional hospital and RHC, and consequent unsafe and inadequate care has been previously reported in this population [4]. Informed by our earlier research, the DoH attempted to improve the maternal discharge summary process. Improvement measures included: redesigning discharge paperwork, nomination of a designated health professional at the RHC to receive summaries and allocate them to the appropriate clinician, hospital staff training in the computer program that generated discharge summaries, and the development of a length of stay and discharge policy [39, 41]. However, numerous examples of compromised patient safety within the infant health system still occurred and were identified by participants. Issues with computer generated discharge summaries [42] were evident in the subsequent sub-study, described here, particularly the timely transfer and accuracy of information. The impact of inadequate discharge information processes on quality of care and patient safety has previously been described [43].

A persistent problem for remote living Aboriginal infants [44, 45], documented previously in the study sites [3] is the high prevalence of growth faltering and anaemia. We found that some staff appeared to accept anaemia and growth faltering in this population as a ‘normal’ part of child growth and development. A potential explanation for the acceptance of these conditions as normal and subsequent failure to initiate treatment, is clinical inertia (CI).

Clinical Inertia has been defined as a failure to start or intensify treatment [46]. Widely recognised as a key factor in the poor management of chronic disease by clinicians [46–48] the three main contributing causes for CI were identified in our study. Clinician factors include poor knowledge and training while patient factors involve distrust of the clinician and poor communication between clinician and patient [48]. Health system factors include a lack of guidelines and poor staff communication [48]. Insufficient knowledge and training was acknowledged by participants who described themselves and some of their colleagues as ill prepared for the CFHN role. While guidelines did exist, clinician’s failure to follow these was identified in our earlier research [4] and despite improvement efforts at both the RHC and systems level, continued to be an issue in this study. A possible explanation for the continued poor adherence to guidelines across both studies may be staff ‘burnout’.

Burnout is a syndrome of emotional exhaustion, depersonalisation and reduced personal accomplishment, which can occur among individuals who work with people [49]. Increased levels of employee stress and burnout [50] foster workplace disengagement and an inability to meet work demands, negatively effecting health care quality [50]. Data collection for this study did not measure burnout. However, the RAN workforce reports significantly higher emotional exhaustion than other nursing samples [50] and is a key reason RANs leave their positions [51].

A contributing factor to potential burnout in our study was the unrealistic responsibilities and demands placed on largely unqualified and untrained staff who, without the requisite skills, were expected to deliver the well-child program. The effect of this was demonstrated by the obvious distress of participants recounting their experiences working in the baby-rooms.

Despite one-third of all RHC presentations being for child health and non-acute interventions [3], participants reported an overwhelming focus by staff on acute service provision, at the expense of primary health care programs. The detrimental outcomes on care quality when clinicians working in RHCs experience burnout including, inadequate monitoring and management of chronic conditions and failure to provide preventative health programs have been reported elsewhere [52].

Possible explanations for an acute care focus may also be an emphasis in undergraduate nursing curricula on acute care and limited exposure to, and understanding of primary health care practice and child and family health nursing [53]. As well, participants acknowledged that their postgraduate experience was in more acute areas such as intensive care or emergency nursing.

Dissatisfaction with management practices by RHC managers has previously been reported [51]. Clinicians expressed disappointment *‘at the mediocre management skills which lead to some staff refusing to actually cover areas outlined in business plans’* [51] [p 114] [54]. In our data this was exemplified by participant descriptions of the actions of a RHC manager, employed prior to data collection, who attempted to incorporate the well-child program into general RHC functions because so few staff expressed interest with working in the baby-room, rather than prioritise the program and recruit and stabilise the workforce. This directly contrasts with a key recommendation for Aboriginal child health that:

*“Workforce requirements to provide appropriate services for these children must be prioritised so that the gap in services between them and other children is minimised, not exacerbated”* [55].

The United Nations recognises the rights of children ‘to the highest attainable standard of health’ [54] with an emphasis on primary health care, child health and preventative health care [54]. However, the well-child health program available to the wider Australian population is not offered in remote communities. Systems need to be found to ensure that children in remote areas receive child health visits, as they stand to derive the greatest benefit from child health services’ [55] [p218]. However the inverse care law, predominant within health systems operates in the two study sites, whereby those who need care most are less likely to get it [56]. The child health education package was developed to train the existing workforce to provide a well-child health program, in the absence of qualified staff. While there is support for this approach, it creates an uneasy tension as the use of an unskilled workforce undermines the efficacy of preventative child health programs [55] and a compelling argument that the infant population with the greater disease burden is given an inferior service. The damaging effects of inadequate nurse preparation for preventative health programs is well known [29, 52, 53, 57]. and was recognised by study participants. Preventative health programs are supplanted by acute care demands while follow up care and management is not given and community members, for whom the service is provided, become disengaged [57]. The use of an unskilled workforce to deliver a well-child health program is in contrast to most Australian jurisdictions which require CFHNs to hold postgraduate qualifications [10, 58].

The disengagement with health services by community members as a direct result of staff turnover is recognised [57]. Staff attrition and rotation through the RHC baby-rooms was highlighted by participants as a major contributing factor to poor continuity of care and service quality. Participants described it as almost impossible to build trust and develop relationships with infants and their families, considered key to effective child and family health practice [59] and to reducing emergency presentations [60]. This finding contrasts with results from the evaluation data of a midwifery continuity of care model introduced in the regional centre in 2009. The launch of the Midwifery Group Practice by the DoH, linked Aboriginal women transferring to the regional centre at any time throughout their pregnancy and for birth with a known midwife. This resulted in improved health system engagement [61]. However, a service evaluation found that it was in the RHC where care was more often likely to be suboptimal [62].

Indigenous involvement in health service delivery is key to its uptake by Aboriginal people [63] and the ability to address racism in health services. Yet Indigenous people comprise only 1% of the Australian Health Workforce [64]. This corresponds with Indigenous workforce figures in other developed nations [65]. Lack of support for Aboriginal staff due to high staff attrition and limited appreciation of the importance of the role of Aboriginal staff has been documented previously [66, 67]. However, it fails to explain why no ACWs were recruited to work alongside CFHNs despite available funding. While Aboriginal staff are considered essential to improving service accessibility and acceptability in MIH care [68], this study found no Aboriginal staff working within child health in the two study sites. The marginalisation or lack of Aboriginal health staff silences Aboriginal voices and contributes to their invisibility allowing both conscious and unconscious racism to permeate [69]. Similar findings have been described in the Australian Public Service [70].

Racism is a key determinant of health for Indigenous people [2, 71] and the experience of racism by Aboriginal people can contribute to poorer health outcomes [72]. Racist attitudes and practices by clinicians are described in the literature [69, 72, 73] and were observed in this sub-study. Studies have shown that where their caregivers are exposed to racism, infants are likely to experience more common child health illnesses and poorer social and emotional wellbeing outcomes [74]. Some staff seem to lack insight into the fact that their behaviours are contributing to the problem and continued disadvantage of Aboriginal clients [69]. For example, in this study caregivers were alienated by staff in the way they were spoken to and treated to such a degree that some staff believed that it affected their RHC attendance and return for follow up treatment. These findings are consistent with the unsafe and distressing experiences of Aboriginal people engaging with the broader NT health system [75].

An area requiring urgent reform in closing the gap in Indigenous health outcomes is the development of a culturally competent workforce [76]. The characteristics of such a workforce have been described elsewhere [11]. While the NT DoH has instituted a cultural security framework [77] there needs to be a rigorous examination of the implementation of this by the DoH [3] to determine if current practices are supporting or hindering Aboriginal health and wellbeing [69]. This may necessitate the development of tools to measure culturally competent care [11].

### Limitations

This study is limited by the relatively brief periods of participant observation, thirty hours in both communities, and the small number of participants. A further limitation is that the findings may be non-generalizable as the study took place in two communities only, however similar problems are reported elsewhere [44, 78–80]. The study is strengthened by using the same methods and methodology as our previously published study [3].

## Conclusion

Australian Aboriginal infants have poorer health outcomes than their non-Aboriginal counterparts. It is concerning that problems such as poor information transfer and poor adherence to departmental guidelines are repeatedly raised as serious issues that affect care quality and influence patient health outcomes but these continue to be substandard. They remained so despite efforts to specifically address these issues [4]. This study relies on data collected between 2009 and 2012. We acknowledge that in the intervening years the DoH may have rectified a number of these issues.

The health system is in itself, a social determinant of health, especially when it fails to meet the requirements of those who most need it [81]. The causal factors for poor quality care in our study included ineffectual service design, poor staff organisation, lack of training and skills required for practice, limited preventative child health experience, deficient continuity of care/carer, few Aboriginal staff and racism. There is an urgent need for better management practices and MIH system reform to improve the quality of care provided to remote-dwelling Aboriginal infants and their health outcomes.

## Additional file

Additional file 1: Description of an encounter between a RAN and a mother observed in a baby room. (DOCX 11 kb)

## Abbreviations

ACW: Aboriginal community worker
AHW: Aboriginal health worker
CFHN: Child and family health nurse
DMO: District medical officer
DoH: Department of Health
EHR: Electronic health record
FTE: Fulltime equivalent
GP: General practitioner
HU5K: Healthy Under 5 Kids
MGP: Midwifery group practice
MIH: Maternal and infant health
MO: Medical officer
NT: Northern Territory
PAR: Participatory action research
RAN: Remote area nurse
RHC: Remote health centre
TE: Top end

## Footnotes

1 There is no consistent terminology applied to nurses who provide community based services in the 0–5 population. In the Northern Territory (NT) these nurses are called child and family health nurses; throughout Australia though, the terminology varies. For example, maternal and child health nurse is used in Victoria.

2 Central Australian Rural Practitioners Association CARPA Standard Treatment Manual – a clinic manual for primary health care practitioners in remote and rural communities in Central and Northern Australia. Remote Health Atlas available online and in hard copy to provide governance and information for staff. It sets standards and provides guidance regarding the DoH remote health branch, policy and protocol.

3 A term referring to non-Aboriginal people in the TE of Australia.
