# Supplementary material for: The quality of health services provided to remote dwelling aboriginal infants in the top end of northern Australia following health system changes: a qualitative analysis
Source: BMC Pediatr. 2017 Mar 31;17:93. doi: 10.1186/s12887-017-0849-1 (PMC5374585; doi:10.1186/s12887-017-0849-1)
Supplement: Additional file 1: — Description of an encounter between a RAN and a mother observed in a baby room. (DOCX 11 kb) [file 12887_2017_849_MOESM1_ESM.docx]

The RAN called a woman into the room. Eileen (not her real name) entered the room with her child following behind her and the RAN said ‘what mother leaves her child and comes in on her own’. Eileen picked her child up by the arm and the RAN spoke to her very crossly saying ‘don’t pick her up like that’ suggesting that Eileen could dislocate the child’s arm picking her up that way. She then asked ‘what is child’s name?’ and Eileen responded ‘Mary’. The RAN said ‘Mary Jane?’ and Eileen said ‘no just Mary’. The RAN looked at the computer and rolled her eyes saying ‘there is Mary Jane here – don’t you know your own child’s name?’ The RAN then admonished Eileen saying ‘this child was meant to be weighed every week – why haven’t you brought her?’ Eileen's friend walked past the room and Eileen called out to her in Aboriginal language. The RAN said ‘you can talk to her later – listen to me now, talk to me listen to what I am saying’. Eileen started to breastfeed her child and the RAN said ‘stop breastfeeding this one you have another one on the way’ (Observation Community B July 2012).
